# Acute kidney injury during pregnancy and puerperium: a retrospective study in a single center

**DOI:** 10.1186/s12882-017-0551-4

**Published:** 2017-05-01

**Authors:** Chunhong Huang, Shanying Chen

**Affiliations:** Department of Nephrology, Zhangzhou Affiliated Hospital of Fujian Medical University, Zhangzhou, 363000 People’s Republic of China

## Abstract

**Background:**

Acute kidney injury (AKI) is rare in women during pregnancy and puerperium, however, it is related to increased morbidity and mortality rates.

**Objective:**

The aim of this study was to investigate the incidence, characteristics, and outcomes of AKI during pregnancy and puerperium in a Chinese population.

**Methods:**

In this study, pregnant women discharged from hospital between January 2008 and June 2015 were screened. AKI was defined if the level of serum creatitine >70.72umol/l in pregnant women without chronic kidney disease (CKD). Acute-on-CKD was defined as a 50% increase in the level of serum creatinine vs baseline in patients with pre-existed CKD.

**Results:**

We reported a high incidence (0.81%) of AKI during pregnancy and puerperium. Three hundred and forty-three cases of AKI during pregnancy and puerperium included 21 severe AKI cases and 21 cases with acute-on-CKD. Pre-eclampsia/eclampsia, and postpartum hemorrhage were the most frequent causes of AKI during pregnancy and puerperium. About 17% women with pre-eclampsia/eclampsia and 60% women with HELLP syndrome complicated with AKI. The maternal outcome was good except in the setting of amniotic fluid embolism or hemorrhagic shock, whereas the prenatal outcome was relatively poor. Among the 14 death cases, 7 cases received renal replacement therapy. Amniotic fluid embolism and postpartum hemorrhage were the major causes of death in pregnant women with AKI.

**Conclusion:**

AKI during pregnancy and puerperium is not as rare as we thought. Pre-eclampsia/eclampsia is the most common cause of AKI during pregnancy and puerperium, however, the outcome of pre-eclampsia-related AKI is good. Amniotic fluid embolism and postpartum hemorrhage are the leading causes of maternal mortality. Severe AKI may predict poor outcome.

**Electronic supplementary material:**

The online version of this article (doi:10.1186/s12882-017-0551-4) contains supplementary material, which is available to authorized users.

## Background

According to the database from National Maternal and Child Mortality Surveillance System in China, maternal mortality rate was 24.5 per 100,000 live births in 2012 [1]. Acute kidney injury (AKI) is rare in women during pregnancy and puerperium, however, it is related to increased morbidity and mortality rates [2]. The incidence of acute renal failure during pregnancy has declined in both developed and developing countries [3, 4]. Compared with developed countries, China’s maternal mortality rate is relatively high [5].

It is considered that AKI has become a rare complication of pregnancy [4, 6]. Since the 1970s, its incidence in industrialized countries has decreased dramatically because of the disappearance of septic abortion and improved prenatal care [6]. However, there is a paucity of data on pregnancy-related AKI in Chinese women. In a previous review [7], 29 articles about pregnancy-related AKI between 1984 and 2013 were included. These studies included a total of 708 Chinese women with pregnancy-related AKI. The incidence of pregnancy-related AKI in different provinces in China ranged from 0.02% to 1.84% [7]. Much of the data were from small case series. [7]. In a retrospective study in China [8], 75 out of 2988 (2.51%) pregnant women were diagnosed with acute renal insufficiency. In this Chinese population, the incidence of pregnancy-related AKI is much higher than in developed countries [4]. A potential explanation is that a more sensitive criterion was used to diagnose renal insufficiency in the study. In this population, 57 cases with renal insufficiency were defined as mild renal insufficiency (serum creatitine 70–123 umol/L), and only 6 patients were diagnosed with severe renal insufficiency (serum creatitine >221 umol/L) [8]. Considering China’s huge population, a sample of 2988 pregnant women was a relatively small sample size. Because AKI is associated with an increased rate of maternal death [2], the characteristics of AKI during pregnancy and puerperium is worth investigating. We conducted this retrospective study to investigate the incidence, clinical characteristics, etiology and outcome of AKI during pregnancy and puerperium in a large Chinese population.

## Methods

### Patients

This was a retrospective study in a single center. We used an inpatient database from Zhangzhou Affiliated Hospital of Fujian Medical University. The inpatient database contained information on patient’s name, age, gender, and diagnosis. Patients who discharged from January 2008 to June 2015 were screened in accordance with the following diagnoses: 1. Pregnancy or puerperium; 2. Acute renal failure, acute renal insufficiency, or acute kidney injury; (In Chinese language, we use the same character to indicate renal and kidney). A review of complete medical records confirmed the diagnosis of AKI.

A recent study in China reported a high rate of missed and delayed diagnosis of AKI in both academic and local hospitals [9]. Diagnostic information could not accurately reflect the true incidence of AKI. Electronic medical records have included admission notes, on-service notes, case notes and discharge reports since 2008 in our hospital. Laboratory reports were also included in the electronic medical records from May 2011. Using the inpatient database from January 2008 to June 2015, we screened pregnant women with pre-eclampsia/eclampsia, heart failure, shock, pyelonephritis, pregnant fatty liver, postpartum hemorrhage, and amniotic fluid embolism. Above diseases are associated with an elevated incidence of AKI during pregnancy and puerperium [7]. We reviewed detailed electronic medical records to confirm a diagnosis of AKI. We also reviewed the pregnant cases with multiple organ dysfunction syndrome (MODS). If the medical records of the cases with MODS indicated kidney injury, these cases were also included in the present study.

### Definition

According to the introduction by KDIGO, AKI is defined if an increase in serum creatitine by ≥26.5 μmol/l within 48 h [10]. However, during gestational period, increased renal blood flow leads to glomerular hyperfiltration accompanying by a lower serum creatinine level. Values considered normal in the non-pregnant state may be abnormal during pregnancy [11]. In the present study, AKI was defined if the level of serum creatitine was >70.72umol/l in pregnant women without chronic kidney disease (CKD) [12]. Chronic kidney disease was defined as presence of any indicators for kidney disease including declined renal function (GFR < 60 ml/min/1.73m^2^), proteinuria, hematuria and/or renal pathological leisure at least three months [13]. In CKD patients, AKI was defined as a 50% increase in the level of serum creatinine vs baseline [10]. This was defined as acute-on-CKD. If a woman had persistent kidney injury, we checked her medical records from pre-pregnancy to the first trimester to exclude CKD. Severe AKI was defined as receiving renal replacement therapies. In a previous study based on a relatively large population in China, severe renal failure was defined as serum creatitine >221 umol/L [8]. For purposes of comparison, we also listed the number of patients with the levels of serum creatitine >221umol/L.

Pre-eclampsia was defined as a set of two signs: proteinuria and hypertension after 20 weeks of gestation. Hypertension was defined as systolic blood pressure ≥ 140 mmHg and/or a diastolic blood pressure ≥ 90 mmHg. Eclampsia was defined as existence of pre-eclampsia and seizures. HELLP syndrome was defined as existence of pre-eclampsia, hemolysis, elevated liver enzymes and thrombocytopenia [14]. Hemolytic uremic syndrome (HUS) was defined as existence of hemolytic anemia, AKI and thrombocytopenia. Postpartum hemorrhage was defined as bleeding more than 500 ml within the first 24 h following delivery [15].

The first trimester was defined from conception to the 13th week. The second trimester was referred to conception from the 14th until the 28th week. The third trimester was referred to conception from the 28th week until delivery. Puerperium was defined as 6 weeks post-delivery or post-abortion [16].

### Maternal and fetal Outcome

We assessed the maternal outcome of AKI and also calculated the maternal mortality.

Term delivery was defined as the birth of a neonate after 37th week of gestation [17]. A stillbirth was defined when a fetus died in the uterus after 20 week of gestation [18]. A small-for-date newborn infant was referred to a newborn infant that was born after 37th week of gestation and weight was <2500 g [17].

### Data analysis

We analyzed the incidence and the causes of AKI during pregnancy and puerperium. The incidences of AKI in patients with pre-eclampsia/eclampsia, HELLP syndrome, pregnant fatty liver, or postpartum hemorrhage were also calculated. According to the onset time of AKI, pregnant women with AKI were divided into four groups: group-1: AKI during the first trimester; group-2: AKI during the second trimester; group-3: AKI during the third trimester and group-4 AKI during puerperium. Characteristics of patients in four groups were listed. Continuous variables were shown as mean ± standard deviation or median and quartile according to the distribution of the variables. The categorical variables were presented as absolute and/or relative (%) values.

We also investigated the characteristics, therapies and outcomes of pregnant women with severe AKI. Data analysis was conducted using Stata software (Version 12.0).

## Results

During the study period (7.5 years), among 42,173 cases discharged from obstetrics department, 343 cases had evidence of AKI. A flowchart describing the selection of patients was filed (Additional file 1). Only 67 cases (19.53%) with AKI were diagnosed with acute renal failure, acute renal insufficiency, or acute kidney injury during hospitalization. About 80 % of the cases (276/343) had been misdiagnosed. The incidence of AKI during pregnancy and puerperium was 0.81% (about 1 to 123). Among 42,173 pregnant cases, the cases with pre-eclampsia/eclampsia, postpartum hemorrhage, pregnant fatty liver, and HELLP syndrome were 1427, 837, 55 and 53, respectively. The incidence of AKI in women with pre-eclampsia/eclampsia was 16.82% (240/1427). The incidences of AKI in women with postpartum hemorrhage, pregnant fatty liver, and HELLP syndrome were 4.78% (40/837), 20% (11/55), and 60.38% (32/53), respectively. Fourteen dead cases were reported and the maternal mortality was 4.08% among pregnant women with AKI.

### Characteristics of women with AKI (Table 1)

Three hundred and forty-three pregnant women with a mean age of 29 years were included in the present study. Most cases (79.3%) developed AKI during the third trimester of pregnancy. Two hundred and ninty-one patients had at least one pregnant complication. The most common cause of AKI during pregnancy and puerperium was pre-eclampsia/eclampisa followed by postpartum hemorrhage. In addition, 16 cases with AKI during the third trimester of pregnancy were concomitant with postpartum hemorrhage which may exacerbate kidney injury. Hemorrhage of ectopic pregnancy rupture was the leading cause of AKI during the first trimester of pregnancy. Eight patients with pregnant complication had pneumonia. In these cases, infection was unlikely the etiology of AKI, however, it might play a synergistic role in the occurrence of AKI.

**Table 1:** ^a^Basic information of 343 pregnant women with acute kidney injury

	Total	Group 1	Group 3	Group 3	Group 4
	*N* = 343	*N* = 12	*N* = 24	*N* = 272	*N* = 35
Age (years)	29.41 ± 5.94	30.50 ± 5.79	29.58 ± 6.08	29.47 ± 6.05	28.40 ± 5.14
Gestational weeks upon admission(weeks)	33.43 ± 6.68	7.70 ± 1.29	23.28 ± 3.25	35.00 ± 3.18	38.33 ± 2.36
Primipara (%)	179 (52.19)	6 (50)	13 (54.17)	147 (42.86)	13 (37.14)
Twin pregnancy	52 (15.16)	0	0	47 (17.28)	5 (14.29)
Triplet pregnancy	4 (1.17)	0	0	4 (1.47)	0
Blood urea nitrogen (umol/L)	8.73 ± 7.03	7.38 ± 3.76	11.58 ± 6.15	7.84 ± 5.91	14.22 ± 11.97
Serum creatitine(mmol/L)	148.57 ± 142.41	127.15 ± 72.91	243.74 ± 175.15	128.73 ± 123.20	243.77 ± 204.44
Serum creatitine > 221 mmol/L	48 (13.99)	1 (8.3)	9 (37.5)	25 (9.19)	13 (37.14)
Pregnant complications	291 (84.84)	10 (83.33)	10 (41.67)	240 (88.24)	31 (88.57)
Pre-eclampisa	226 (65.89)	0	8 (33.33)	212 (77.94)	6 (17.14)
Eclampisa	14 (4.08)	0	1 (4.17)	12 (4.41)	1 (2.86)
HELLP syndrome	32 (9.33)	0	3 (12.5)	29 (10.66)	0
Postpartum hemorrhage	40 (11.66)	0	0	16 (5.88)	24 (68.57)
Ectopic pregnancy	10 (2.92)	10 (83.33)	0	0	0
Amniotic fluid embolism	6 (1.75)	0	0	1 (0.37)	5 (14.86)
Pregnancy fatty live	11 (3.21)	0	0	10 (3.67)	1 (2.86)
Peripartum cardiomyopathy	3 (0.87)	0	0	3 (1.10)	0
Gestational diabetes mellitus	16 (4.66)	0	1 (4.17)	15 (9.19)	0
Postpartum retention of urine	2 (0.58)	0	0	0	2 (5.7)
Comorbidities					
Chronic kidney disease	21 (6.12)	1 (8.33)	8 (33.33)	12 (4.40)	0
Primary heart diseases	5 (1.46)	0	1 (4.17)	4 (1.47)	0
Obstructive nephropathy	4 (1.17)	0	4	0	0
Pyelonephritis	6 (1.75)	0	5 (20.83)	0	1 (2.86)

^a^Group-1: AKI during the first trimester; group-2: AKI during the second trimester; group-3: AKI during the third trimester and group-4 AKI during puerperium

In the comorbidities, CKD was the leading cause of AKI during pregnancy and puerperium. In addition to CKD, primary heart disease was another cause of AKI during pregnancy. Five patients with heart diseases including congenital atrial septal defect, congenital ventricular septal defect, tetralogy of Fallot, primary pulmonary hypertension and dilated cardiomyopathy developed heart failure and AKI during pregnant. Infectious diseases led to AKI included pyelonephritis, severe pneumonia, cellulitis and epidemic hemorrhagic. Diabetes ketoacidosis led to two cases of AKI in the present study. Other rare causes of AKI included severe acute pancreatitis, acute cerebral accident unrelated to pregnancy, severe thalassaemia with anemic cardiopathy and so on.

### Outcome of AKI during pregnancy and puerperium

Fourteen death cases were reported in this population and the maternal mortality was 4.08% (Table 2). Amniotic fluid embolism and postpartum hemorrhage were leading causes of maternal mortality in pregnant women with AKI. Hemorrhagic shock from a ruptured ectopic pregnancy led to two death cases. Because in most death cases AKI occurred in the postpartum period, the outcome of perinatal infants was relatively good and nine women had live births.

**Table 2:** List of death cases

Case No	The cause of death	Renal replacement therapy	Prenatal outcome
1	Hemorrhagic shock from a ruptured ectopic pregnancy	No	
2	Acute cerebral vascular accident	No	Stillbirth
	(Right cerebellar hemorrhage)		
3	Amniotic fluid embolism	Yes	Live birth
4	Postpartum hemorrhage, hemorrhagic shock	No	Live birth
5	Amniotic fluid embolism	Yes	Live birth
	Postpartum hemorrhage		
6	HELLP syndrome	No	Live birth
7	Amniotic fluid embolism	Yes	Live birth
	Postpartum hemorrhage hemorrhagic shock		
8	AFLP, MODS	Yes	Live birth
9	Hemorrhagic shock from a ruptured ectopic pregnancy	Yes	
10	Postpartum hemorrhage	Yes	Live birth
	Amniotic fluid embolism		
11	Postpartum hemorrhage, MODS	Yes	Live birth
12	Primary pulmonary hypertension	No	Live birth
	Heart failure,preeclampsia		
13	Amniotic fluid embolism	No	Neonatal death
14	Amniotic fluid embolism	No	Stillbirth

Most patients with AKI achieved completely recovery. One case with pre-eclampsia had an elevated serum creatitine level (157.1umol/L) and proteinuria one months after delivery. One woman with pre-eclampsia and peripartum cardiomyopathy had persist proteinuria and hypertension. Three years after delivery, her serum creatitine increased to 342umo/L.

### Perinatal outcome in AKI during pregnancy and puerperium (Table 3)

Fifty-two women with twin pregnancy and 4 women with triplets pregnancy were included in the present study. Ten women with ectopic pregnancy had no data of fetuses and outcomes of 13 fetuses were missing. In the present study, data on 380 perinatal infants were included. The mortality of perinatal infants was 17.11% (65/380). Perinatal outcome of pregnant women with AKI during the second trimester was worse than the third trimester of pregnancy (*P* < 0.05).

**Table 3:** Perinatal outcome of 343 pregnant women with acute kidney injury

	Total	Group 1	Group 2	Group 3	Group 4
Number of pregnant women	343	12	24	272	35
Twin pregnancy	52	0	0	47	5
Triplet pregnancy	4	0	0	4	0
Ectopic pregnancy	10	10	0	0	0
Loss to follow-up	13	1	2	8	2
Number of prenatal	380	1	22	319	38
Death (%)	65	1	17	43	4
Birth weight, g	2209 ± 1429	450	1072 ± 966	2236 ± 1461	2702 ± 606
Birth weight > 2500 g (%)	95	0	3	82	10
Apgar scores					
1 min	9 (6–9)		0 (0–0)	9 (7–9)	9 (7–9)
5 min	10 (8–10)		0 (0–0)	10 (9–10)	10 (8–10)
10 min	10 (8–10)		0 (0–0)	10 (9–10)	10 (10–10)
Delivery by cesarean section	268	0	2	242	24
Term delivery (>37 weeks)	132	0	2	106	24

### Pregnant women with severe AKI (Table 4)

Only 21 patients with AKI received renal replacement therapy. The causes of severe AKI included pre-eclampsia/eclampsia, amniotic fluid embolism, postpartum hemorrhage and so on. Although the incidence of severe AKI was low (6.10%), the outcome was poor. The maternal mortality was 33.33% (7/21) in pregnant women with severe AKI. Case 10 was diagnosed with AKI, severe pre-eclampsia, peripartum cardiomyopathy, and acute heart failure. She discharged with a normal range of serum creatitine, however, she had persistent hypertension and proteinuria after delivery. She was lost to follow-up in department of nephrology. Three years after delivery, the level of serum creatitine increased to 342umol/L. A patient with IgA nephropathy (case 16) had hypertension, mild proteinuria and a normal level of serum creatitine before pregnancy. During pregnancy, her proteinuria and blood pressure increased, however, she declined therapy until the emergence of acute heart failure. After receiving renal replacement therapy, heart failure was controlled. Her renal function did not recover, so she had to receive maintain dialysis therapy. Case 21 was diagnosed with infection-induced HUS. The patient gave a history of fever and diarrhea. Both blood and bone marrow cultures were positive for *E. coli*. She referred to another hospital, so the outcome of her renal function was missing.

**Table 4:** Characteristics of women receiving renal replacement therapy

Case No	Etiology of AKI	Renal replacement	Maternal outcome	Prenatal outcome
1	Tetralogy of Fallot, heart failure	CVVH	Recovery	Premature infant
2	Preeclampsia, placental abruption	HDF	Recovery completely	Stillbirth
3	Epidemic hemorrhagic fever	HD	Recovery completely	Loss follow up
4	Chronic kidney disease	HD	ESRD	Induced labor
5	Amniotic fluid embolism	CVVH	Death	Term infant
	Postpartum hemorrhage			
6	Preeclampsia, pneumonia	CVVH	Recovery	Premature infant
7	Amniotic fluid embolism	CVVH	Death	Term infant
	Postpartum hemorrhage			
8^a^	AFLP	CVVH + PE	Recovery completely	Small-for-date infant
9	Postpartum hemorrhage	CVVH	Recovery	Without data
10	Preeclampsia, peripartum cardiomyopathy, heart failure	CVVH	Chronic kidney disease	Premature infant
11	Postpartum hemorrhage	CVVH	Recovery	Term infant
12	HELLP	CVVH + PE	Recovery	Stillbirth
13	AFLP	CVVH + PE	Recovery	Stillbirth
14	Postpartum hemorrhage,shock	CVVH	Death	Premature infant
15^a^	HELLP	CVVH	Recovery completely	Stillbirth
16	Chronic kidney disease	CVVH	ESRD	Stillbirth
17	AFLP, MODS	CVVH + PE	Death	Term infant
18	Hemorrhagic shock from a ruptured ectopic pregnancy	CVVH	Death	
19	Postpartum hemorrhage	CVVH + PE	Death	Term infant
	Amniotic fluid embolism			
20	Postpartum hemorrhage, MODS	CVVH	Death	Term infant
21	HUS post *Escherichia coli* infection	CVVH + PE	Improved condition	Term infant

^a^Twin pregnancy; *AFLP* fatty liver of pregnancy, *MODS* Multiple organ dysfunction syndrome, *HUS* hemolytic uremic syndrome, *CVVH* continuous veno-venous hemofiltration, *HD* hemodialysis, *HDF* hemodiafiltration, *PE* plasma exchange, *ESRD* end stage kidney disease

### Pregnant women with acute-on-CKD

Twenty one women with CKD developed AKI during pregnancy. Primary kidney diseases included IgA nephropathy, diabetic nephropathy, autosomal dominant polycystic kidney disease, lupus nephritis and so on. Before pregnancy, four patients had an elevated level of serum creatitine (139–377.5 umo/L), and three of them developed ESRD within two years after delivery**.** Prenatal outcome was poor in pregnant women with acute-on-CKD, and only six women got live births.

## Discussion

The merit of the present study is that we used a large sample including 42,173 cases hospitalized in the department of obstetrics. In the present study, the incidence of AKI during pregnancy and puerperium was 0.81%. Twenty one pregnant women received renal replacement therapy. Pre-eclampsia/eclampsia and postpartum hemorrhage were the most common causes of AKI during pregnancy and puerperium. In women with pregnant complications, patients with HELLP syndrome had the highest incidence (60%) of AKI. Maternal mortality in AKI during pregnancy and puerperium was 4.08%, whereas prenatal mortality was higher than maternal mortality (17.11%). Amniotic fluid embolism and postpartum hemorrhage were the leading causes of maternal mortality in AKI during pregnancy and puerperium. Hemorrhage of ectopic pregnancy rupture was the leading cause of maternal mortality in AKI during the first trimester of pregnancy. Pregnant complications play a crucial role in the pathogenesis of AKI.

It has been reported that the incidence of pregnancy-related AKI has declined to 1 in 18,000 in developed countries since the 1960s [4]. In the present study, we found a relatively high incidence of AKI during pregnancy and puerperium. One potential explanation is that we used the low value of serum creatitine (> 70.72umol/L) to define AKI. From early pregnancy, increased renal blood flow leads to an increase in the filtration rate by more than 50% [11]. Changes may persist for up to 12 weeks postpartum. Normal plasma creatinine falls to 44 umol/L and any value above 70.72umol/L should be considered abnormality [11, 12]. Under-recognition of AKI and a delayed diagnosis might lead to underestimate the incidence of AKI during pregnancy and puerperium. In one previous study based on a Chinese population, 70.72umol/L was used as the cut-off value for abnormalities in pregnant women. Seventy-five cases (2.51%) of AKI were identified in 2988 adult pregnant women [8]. In this previous study, 6 (0.20%) cases with severe renal failure (serum creatitine >221umol/l) were reported. In the present study, 48 (0.14%) patients had the level of serum creatitine >221u mol/l. Both the previous study and the present study indicate that AKI during pregnancy and puerperium is not rare and may be underestimated in clinical practice.

In the present study, the major cause of AKI during pregnancy is pregnancy-related complications. Pre-eclampsia/eclampsia is the most frequent cause of AKI during pregnancy. Most AKI occured during the third trimester and the postpartum period. About 17% women with pre-eclampsia/eclampsia were diagnosed as AKI. The result is similar to the previous one [19]. More than 60% cases with HELLP syndrome complicated with AKI. Women with HELLP syndrome had the highest incidence of AKI in women with pregnant complications. Martínez de Ita AL et al [20] reported the incidence of acute renal failure in women with HELLP syndrome was 20%. In this study [20], acute renal failure was considered when serum creatinine was greater than 106.08umol/l for at least 48 h. In the present study, if serum creatinine >106.08umol/l was used to define as acute renal failure, 24.5% (13/53) cases with HELLP syndrome were defined as acute renal failure (not shown in the results).

In most countries, hypertensive complications of pregnancy are the leading cause of pregnancy-related AKI [21]. Pre-eclampsia is considered to have placental insufficiency leading to inadequate oxygen delivery to the placenta and the release of mediators of endothelial injury [22]. The resultant deficiency of vascular growth factor activity may contribute to hypertension, proteinuria, and renal injury [23]. Pre-eclampsia is attributed to an excess of vasoconstrictor over vasodilator impacting systemic circulation which would be expected to lower the glomerular perfusion rate [24, 25]. Examination of the pre-eclampsia reveals conspicuous endothelial cellularity in most cases. Thickening of glomerular capillary walls related to mesangial interposition and prominent subendothelial hyaline deposits leads to glomerular filtration impairment [23, 26].

The maternal outcome of AKI during pregnancy and puerperium is relatively good. In the present study, maternal mortality is 0 in pre-eclampsia/eclampsia women without HELLP syndrome. A large-scale population survey indicated that pre-eclampsia is a marker for an increased risk of subsequent end-stage renal disease (ESRD), but the absolute risk of ESRD in women having history of pre-eclampsia is low [27]. In the present study, one woman with pre-eclampsia and peripartum cardiomyopathy developed CKD after delivery. Although she had no evidence of preexist of CKD, latent kidney disease cannot be excluded.

Septic abortion had been seemed as one of the most cause of AKI during pregnancy and a major public health problem in developing countries [2]. One previous study indicated that septic abortion accounted for 50% cases in the first and 25% cases in the second trimester [28]. However, in the present study, no septic abortion was reported and the leading cause of AKI during the first semester of pregnancy was hemorrhagic shock from ruptured ectopic pregnancy. Other relatively rare causes of pregnancy-related AKI include renal cortical necrosis [29] and thrombotic microangiopathy [30]. No case with renal cortical necrosis was reported in the present study. One patients was diagnosed with verotoxin-associated typical HUS, but not pregnancy-associated HUS.

It is not surprising that the outcome of AKI induced by amniotic fluid embolism was disaster. Another major death cause of pregnant women with AKI was postpartum hemorrhage in the present study. Hemorrhagic shock from ruptured ectopic pregnancy led to two maternal death in this population.

Twenty one cases with AKI received dialysis therapy and the incidence of severe AKI was 1 per 2008 of this population. In previous studies, 16–73.33% pregnant women with AKI needed dialysis treatment. However in these studies, a higher value of serum creatitine was used to confirm the diagnosis of AKI. This might be a potential explanation for high mortality rates and high dialysis rates in previous studies [19, 28, 31, 32]. In the latest study of 1,918,789 pregnancies, 188 (about 1 per 10,000) were complicated by AKI needed dialysis therapy [33]. Compared with this study, it seemed that the incidence of dialysis-requiring AKI in the present study is high. However, the incidence of dialysis-requiring AKI in our hospital cannot represent the real incidence in the total population. In Chinese service medical system, general hospitals as well as maternal and child care service centers supply for health service for pregnant women. Because of lacking dialysis device, maternal and child care service centers cannot treat dialysis-requiring AKI. As the biggest tertiary hospital in Zhangzhou City, we received more patients with pregnant complications. In the present study, we also reported a high maternal mortality rate of AKI needing dialysis therapy. In 14 death cases with AKI, 7 patients received renal replacement therapy. The result indicated that severe AKI may be a strong predictor for poor outcome.

Twenty-one acute-on-CKD were reported in the present study. Two patients received dialysis therapy. Baseline renal function, blood pressure and proteinuria are strong predictors for the outcome of pregnant women with CKD [34]. The risk for the mother and the fetus is reduced and a normal pregnancy can be predicted if *serum creatitine is* < 123.76 umol/l in the absence of arterial hypertension and with low-grade proteinuria in women with nephropathies [34]. In patients with serum creatitine >176.8 umol/l, around 30% of women develop ESRD within one year of delivery [34]. In the present study, four patients with CKD developed ESRD within 2 years after delivery. One IgA nephropathy patient with a normal level of serum creatitine before pregnancy did not get rid of dialysis after delivery. Beside pregnancy, severe heart failure, uncontrolled blood pressure and delayed treatment may contribute to renal damage in this case. The result also indicated that the prenatal outcome of women with acute-on-CKD was poor.

Compared with previous studies based on Chinese populations [7, 8], we screened AKI in a large population, and collected 343 pregnant women with AKI including 21 case with severe AKI and 21 cases with acute-on-CKD. The time span of the database is from 2008 to 2015. Several limitations should be indicated here. First, as a single center retrospective study, the present study is prone to selected biases. This is the most important limitation of the present study. Second, we cannot avoid missing diagnosis completely, because we did not review all of 42,173 pregnant women case by case. Therefore, the true incidence might be underestimated. Third, before May 2011, laboratory reports were not included in the electronic medical records. We only used other medical records including case notes, service notes, admission notes and discharge reports to screen patients discharged from hospital before May 2011. Although, it is required to describe patients’ laboratory results in case notes, imprecise records might still exist. Fourth, we only can check the number of cases discharged from department of obstetrics during the study period and use it as the number of pregnant cases. Some of pregnant women experienced multiple admissions. Considering the large population in the present study, this has little effect on the result of incidence of AKI.

## Conclusion

AKI during pregnancy and puerperium is not as rare as we thought. In the present study, we reported a relatively high incidence (0.81%) of AKI during pregnancy and puerperium. Pre-eclampsia/eclampsia is the most common cause of AKI during pregnancy and puerperium. Patients with HELLP syndrome have the highest incidence of AKI. Amniotic fluid embolism and postpartum hemorrhage are major death causes of pregnant women with AKI. The maternal outcome after therapy is good except in the setting of amniotic fluid embolism and hemorrhagic shock, whereas the prenatal outcome is relatively poor. Severe AKI needing renal replacement therapy might be a strong predictor for poor outcome.

## Additional files

Additional file 1: Flowchart describing the selection of patients. (PDF 73.8 kb)
