# Supplementary material for: Acute kidney injury during pregnancy and puerperium: a retrospective study in a single center
Source: BMC Nephrol. 2017 May 1;18:146. doi: 10.1186/s12882-017-0551-4 (PMC5412057; doi:10.1186/s12882-017-0551-4)
Supplement: Supplementary file 1 — Flowchart describing the selection of patients. (PDF 73.8 kb) [file 12882_2017_551_MOESM1_ESM.pdf]

Flowchart for the selection of patients *

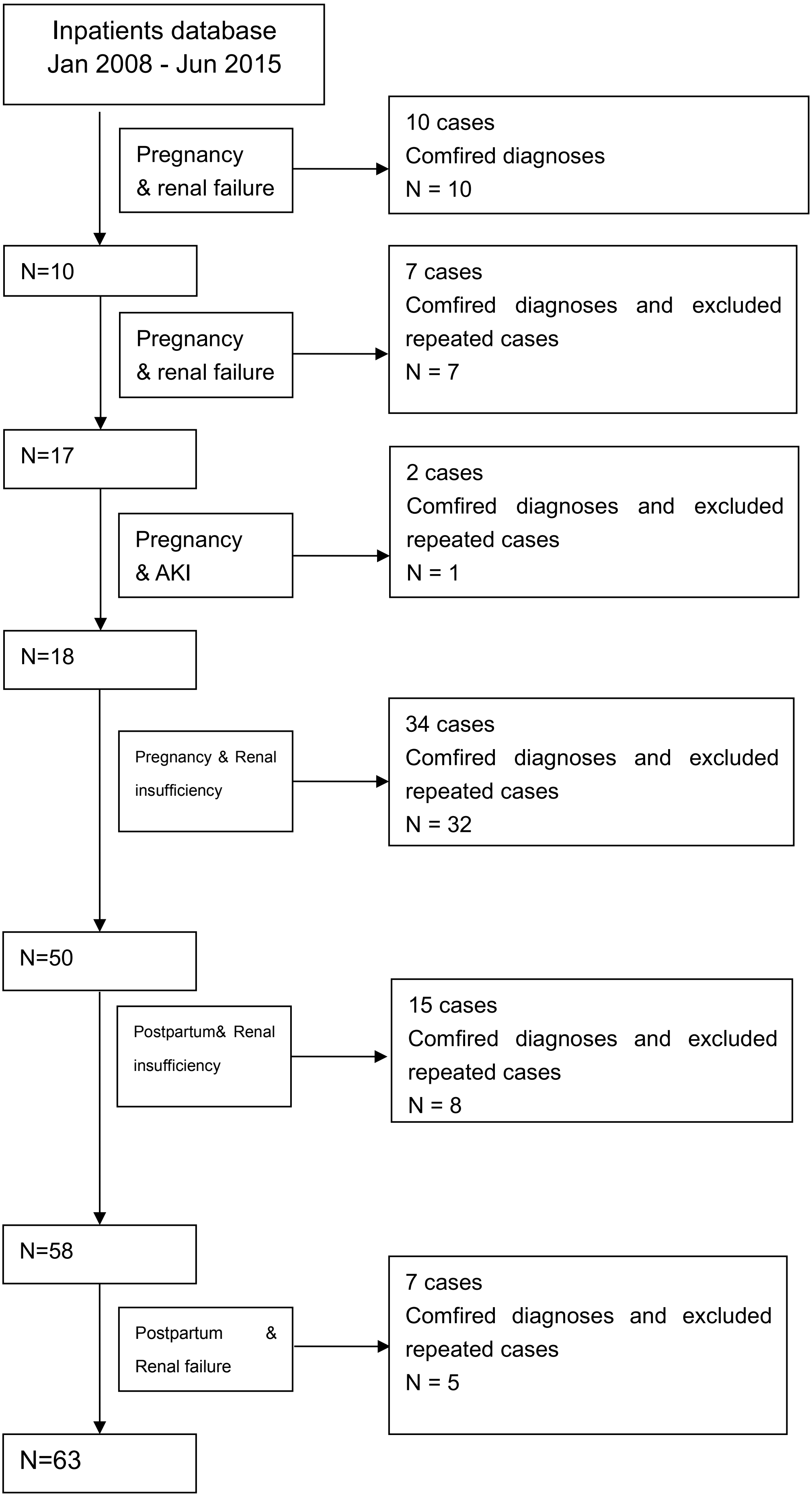

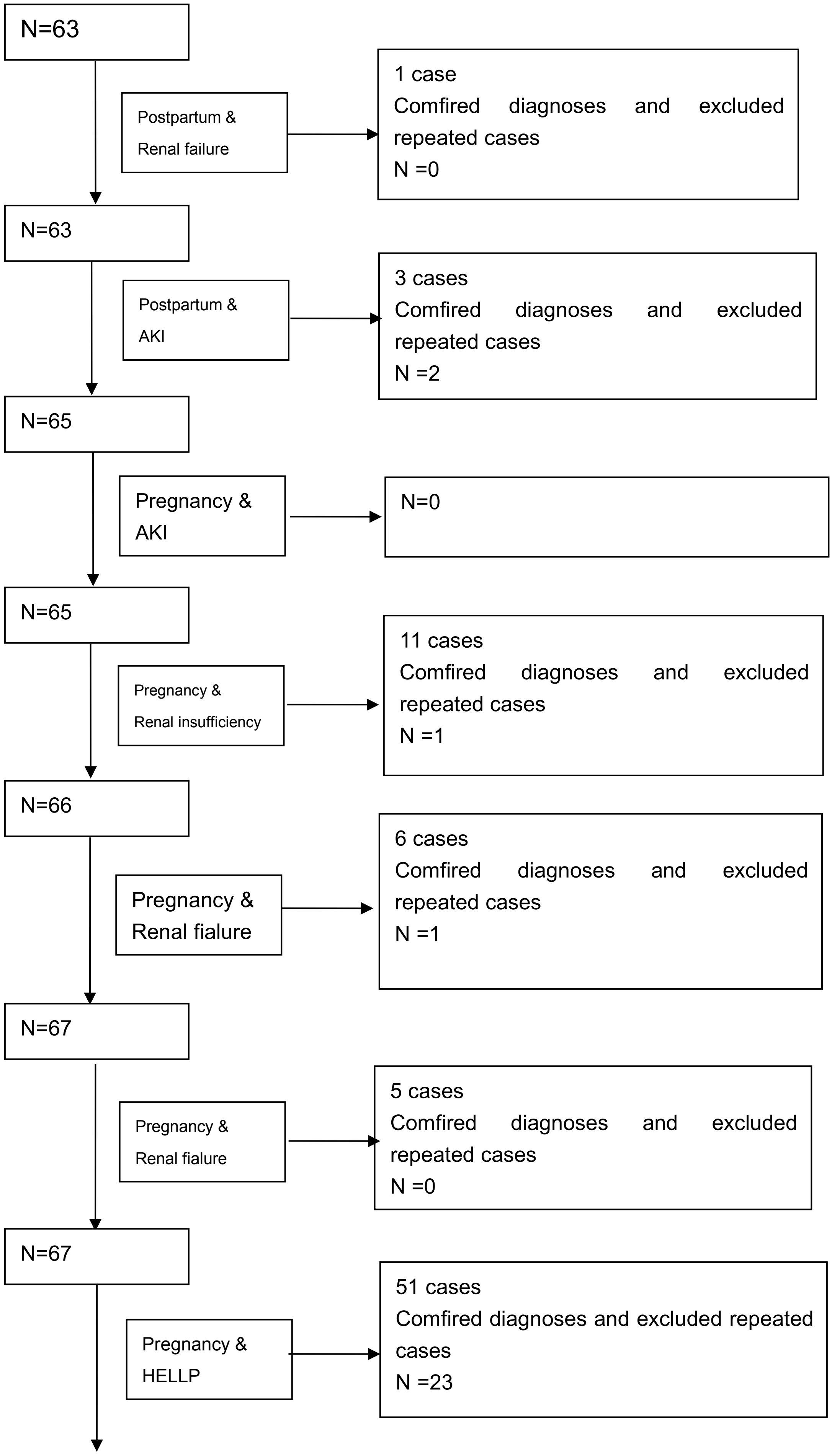
N=63
Postpartum &
Renal failure
1 case
Comfired diagnoses and excluded repeated cases
N =0
N=63
Postpartum &
AKI
3 cases
Comfired diagnoses and excluded repeated cases
N =2
N=65
Pregnancy &
AKI
N=0
N=65
Pregnancy &
Renal insufficiency
11 cases
Comfired diagnoses and excluded repeated cases
N =1
N=66
Pregnancy &
Renal fialure
6 cases
Comfired diagnoses and excluded repeated cases
N =1
N=67
Pregnancy &
Renal fialure
5 cases
Comfired diagnoses and excluded repeated cases
N =0
N=67
Pregnancy &
HELLP
51 cases
Comfired diagnoses and excluded repeated cases
N =23

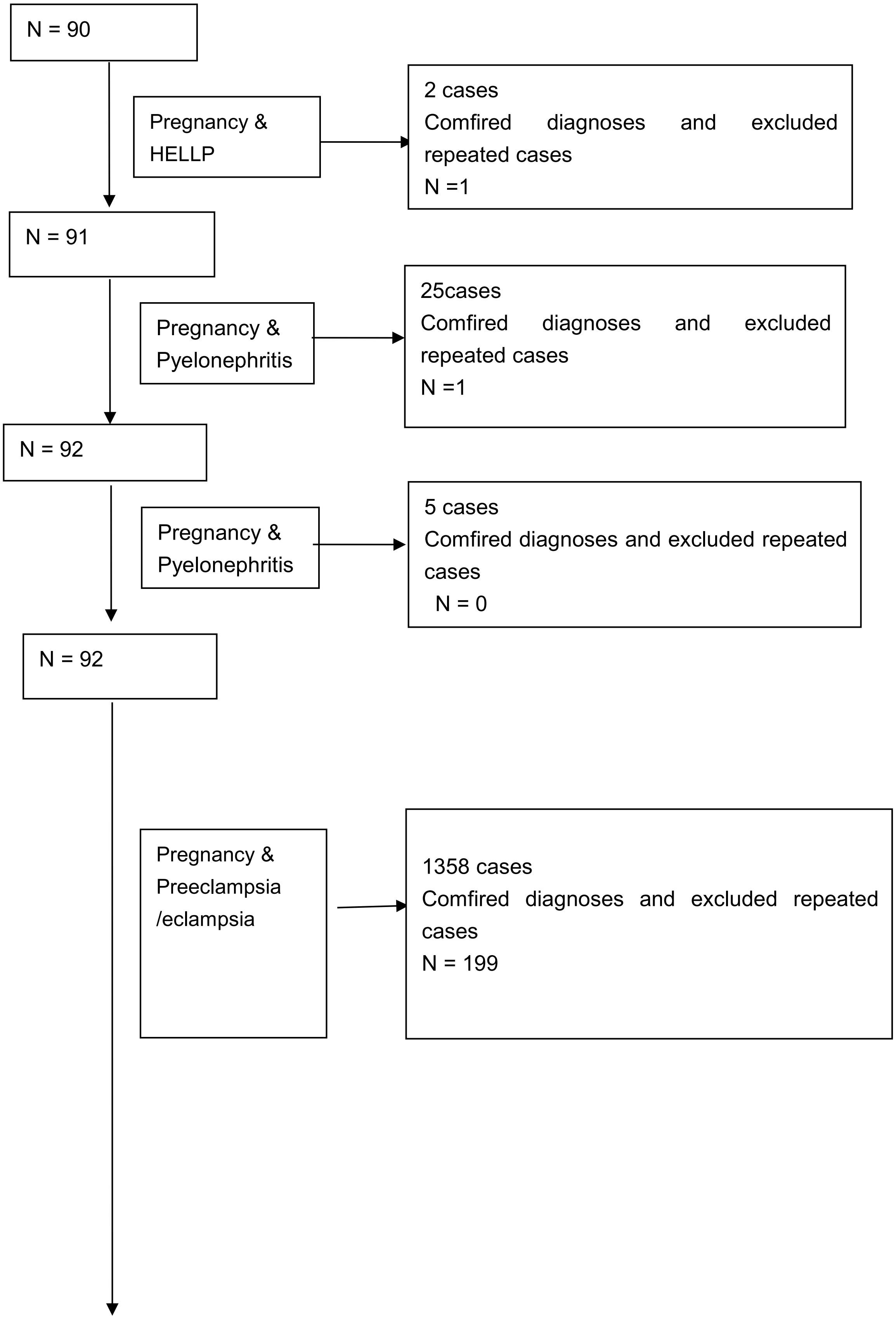
N = 90
Pregnancy & HELLP
2 cases
Comfired diagnoses and excluded repeated cases
N =1
N = 91
Pregnancy & Pyelonephritis
25cases
Comfired diagnoses and excluded repeated cases
N =1
N = 92
Pregnancy & Pyelonephritis
5 cases
Comfired diagnoses and excluded repeated cases
N = 0
N = 92
Pregnancy & Preeclampsia /eclampsia
1358 cases
Comfired diagnoses and excluded repeated cases
N = 199

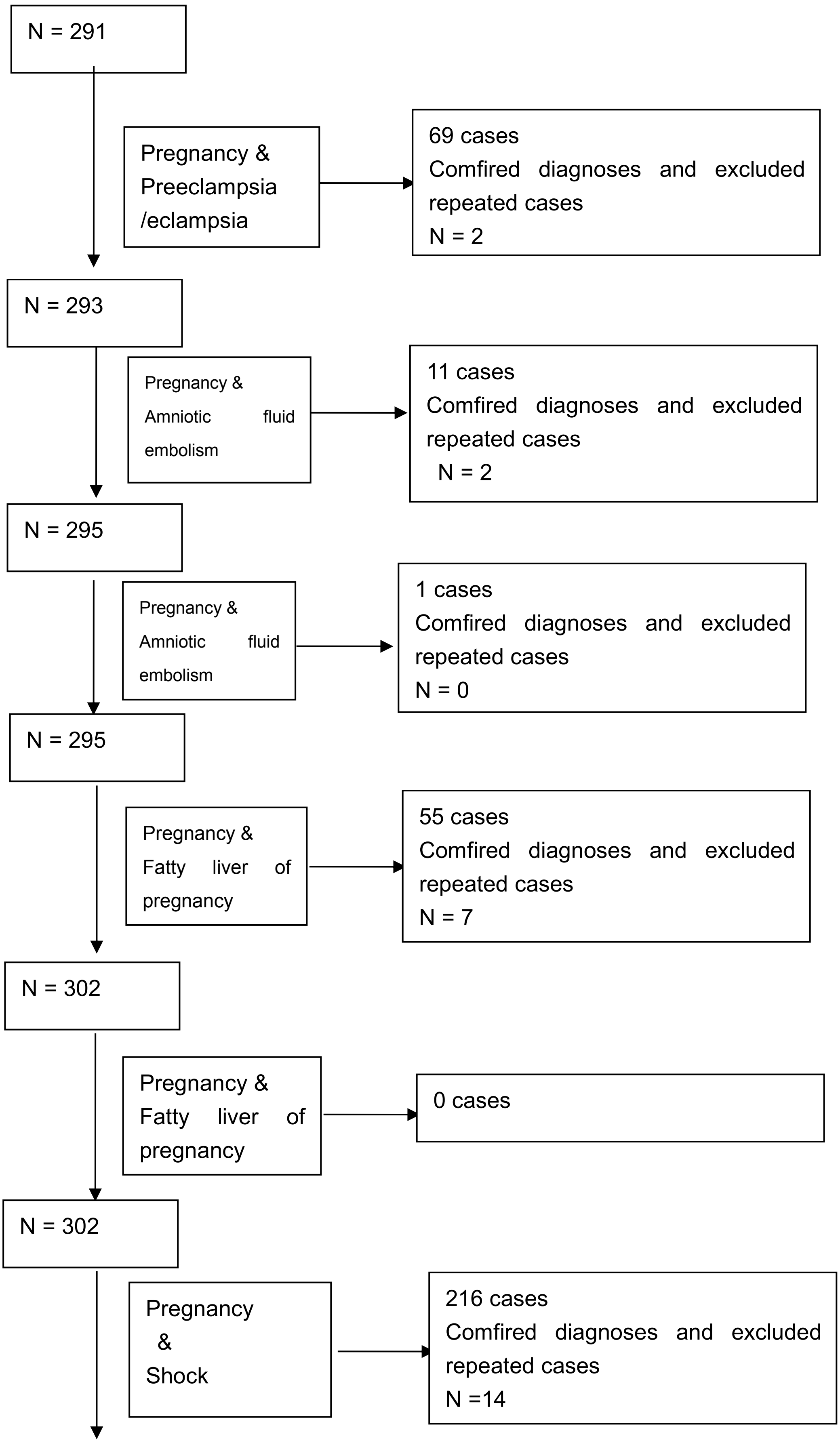
N = 291
Pregnancy & Preeclampsia /eclampsia
69 cases
Comfired diagnoses and excluded repeated cases
N = 2
N = 293
Pregnancy & Amniotic fluid embolism
11 cases
Comfired diagnoses and excluded repeated cases
N = 2
N = 295
Pregnancy & Amniotic fluid embolism
1 cases
Comfired diagnoses and excluded repeated cases
N = 0
N = 295
Pregnancy & Fatty liver of pregnancy
55 cases
Comfired diagnoses and excluded repeated cases
N = 7
N = 302
Pregnancy & Fatty liver of pregnancy
0 cases
N = 302
Pregnancy & Shock
216 cases
Comfired diagnoses and excluded repeated cases
N =14

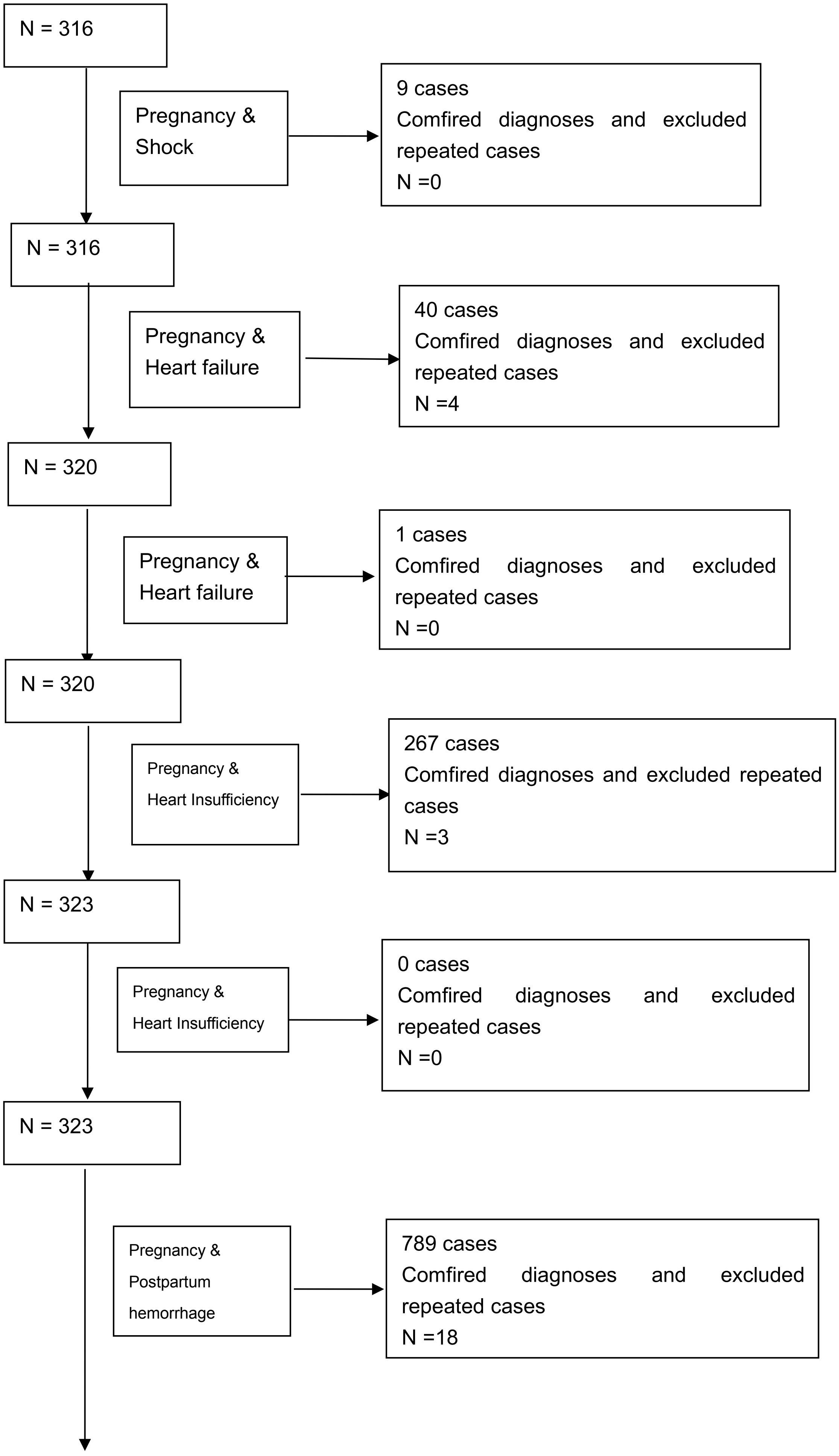
N = 316
Pregnancy & Shock
9 cases
Comfired diagnoses and excluded repeated cases
N =0
N = 316
Pregnancy & Heart failure
40 cases
Comfired diagnoses and excluded repeated cases
N =4
N = 320
Pregnancy & Heart failure
1 cases
Comfired diagnoses and excluded repeated cases
N =0
N = 320
Pregnancy & Heart Insufficiency
267 cases
Comfired diagnoses and excluded repeated cases
N =3
N = 323
Pregnancy & Heart Insufficiency
0 cases
Comfired diagnoses and excluded repeated cases
N =0
N = 323
Pregnancy & Postpartum hemorrhage
789 cases
Comfired diagnoses and excluded repeated cases
N =18

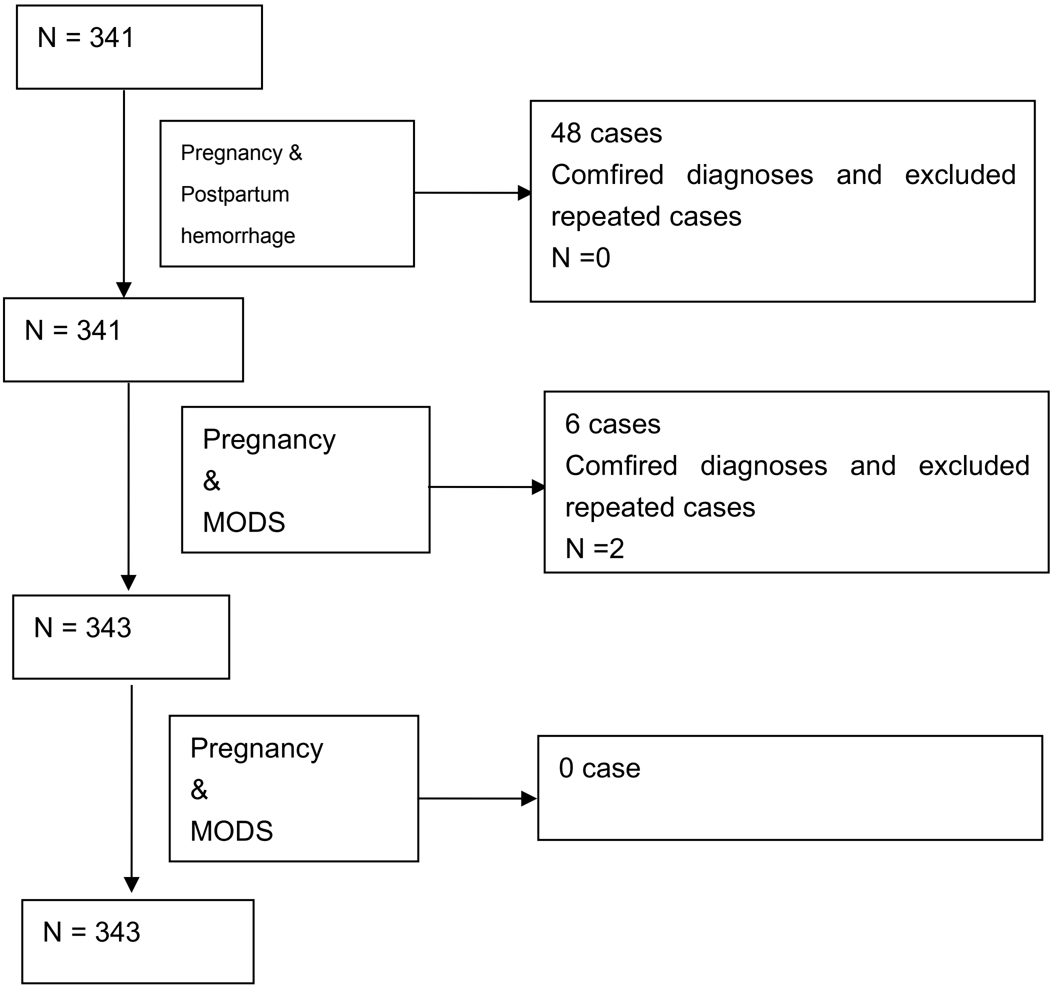

* Two different Chinese characters are used to indicate pregnancy. Both of them were used in the flowchart. Renal failure was also expressed in two ways.
